# Detection of prions in matching *post-mortem* skin and cerebrospinal fluid samples using second-generation real-time quaking-induced conversion assay

**DOI:** 10.1038/s41598-024-56789-6

**Published:** 2024-03-15

**Authors:** Soňa Baranová, Tibor Moško, Magdalena Brůžová, Tracy Haldiman, Chae Kim, Jiri G. Safar, Radoslav Matěj, Karel Holada

**Affiliations:** 1 Institute of Immunology and Microbiology, First Faculty of Medicine, Charles University, Prague, Czech Republic; 2 Department of Pathology and Molecular Medicine, Third Faculty of Medicine, Charles University and Thomayer University Hospital, Prague, Czech Republic; 3 Department of Pathology, Case Western Reserve University School of Medicine, Cleveland, OH 44106 USA; 4 Department Neurology, Case Western Reserve University School of Medicine, Cleveland, OH 44106 USA; 5 Department of Pathology, Third Faculty of Medicine, Charles University and University Hospital Kralovske Vinohrady, Prague, Czech Republic; 6 Department of Pathology, First Faculty of Medicine, Charles University and General University Hospital, Prague, Czech Republic

**Keywords:** Neuroscience, Diseases of the nervous system

## Abstract

Real-time quaking-induced conversion assay (RT-QuIC) exploits templating activity of pathogenic prion protein for ultrasensitive detection of prions. We have utilized second generation RT-QuIC assay to analyze matching *post-mortem* cerebrospinal fluid and skin samples of 38 prion disease patients and of 30 deceased neurological controls. The analysis of cerebrospinal fluid samples led to 100% sensitivity and 100% specificity, but some samples had to be diluted before the analysis to alleviate the effect of present RT-QuIC inhibitors. The analysis of the corresponding skin samples provided 89.5% sensitivity and 100% specificity. The median seeding dose present in the skin was one order of magnitude higher than in the cerebrospinal fluid, despite the overall fluorescent signal of the skin samples was comparatively lower. Our data support the use of *post-mortem* cerebrospinal fluid for confirmation of prion disease diagnosis and encourage further studies of the potential of skin biopsy samples for *intra-vitam* prion diseases´ diagnostics.

## Introduction

Prion diseases or transmissible spongiform encephalopathies (TSEs) are progressive neurodegenerative disorders connected with accumulation of multimeric abnormally folded prion protein (PrP^TSE^) in the central nervous system and to lower extent also in other patient tissues. The PrP^TSE^ propagates by templating the conversion of native prion protein (PrP^C^) conformation to its beta sheet rich pathogenic form^1^.

Early diagnosis of TSEs is challenging given the clinical and molecular TSEs heterogeneity. The most common form of human TSE is sporadic Creutzfeldt-Jakob disease (CJD) which has six main clinical subtypes correlating with the methionine/valine polymorphism at codon 129 of the *PRNP* gene and the size of un-glycosylated isoform of present PrP^TSE^ (MM1, MM2, MV1, MV2, VV1, VV2). In addition, over 30 pathogenic mutations of the *PRNP* gene were linked to different forms of genetic TSEs. Both sporadic and genetic TSEs can be the cause of horizontal transmission of prions e.g. in the context of invasive medical procedures. Diagnostic criteria are based on clinical signs comprising rapidly progressive dementia and diverse neurologic symptoms, but in substantial number of cases the picture is not clear and overlaps with other neurodegenerative diseases. Laboratory analysis includes cerebrospinal fluid (CSF) levels of 14-3-3 and tau proteins, genotyping of *PRNP* gene, electroencephalography and magnetic resonance imaging. However, due to their insufficient sensitivity and specificity, the definitive confirmation of TSE diagnosis relies on *post-mortem* neuropathological evaluation of brain tissue and detection of PrP^TSE^ by immunohistochemistry and/or western blot^2,3^. Advent of detection methods employing templating ability of PrP^TSE^ to change the conformation of natively folded PrP substrate in-vitro are fundamentally changing this situation. The methods are inherently specific as the triggering of the conformational change requires high template sequence similarity with the native substrate. For example β-amyloid or α-synuclein aggregates are not able to trigger the conformational change of PrP substrate^4,5^. In addition, successive cycles of the aggregate growth and template multiplication makes the methods exceptionally sensitive with the limit of PrP^Sc^ detection below 1 fg (10^–15^ g)^6^. This allows detection of PrP^TSE^ in various patient tissues and paves the way for justification of its future utilization for definitive confirmation of TSE diagnosis *intra-vitam*.

Real-time quaking-induced conversion assay (RT-QuIC) uses intensive shaking for breaking up the growing PrP aggregates into fragments which template further PrP aggregation. Thioflavin-T fluorescence is utilized to monitor the growth of aggregates in the real time^7^. The original method was thoroughly optimized to achieve better analytical performance^8^. Using the improved RT-QuIC, 92–97% sensitivity and 100% specificity has been achieved for *ante-mortem* CSF samples in a ring trial^9^. Similar results were obtained with brushing of the olfactory mucosa^10^. These exceptional results led to the implementation of RT-QuIC into revised WHO diagnostic criteria of probable sporadic CJD. However, while lumbar puncture is part of routine diagnostic work-up in patients with clinical suspicion of CJD it is invasive procedure which could be connected with potential complications. The less invasive olfactory mucosa brushing requires trained otolaryngologist and special equipment^11^. Another easily accessible tissue which was shown to harbor prions detectable by RT-QuIC is skin^12,13^, but studies validating its use in human TSE patients are scarce. Initially, Orrú et al. have introduced protocol for skin samples of CJD patients using first generation RT-QuIC assay with recombinant bank vole PrP substrate^12^. Mammana et al. have evaluated the sensitivity and specificity of first generation RT-QuIC assay analysis of CJD skin samples using a simplified protocol utilizing either hamster or bank vole PrP substrate^14^. Recently, Xiao et al*.* have suggested superiority of skin samples in comparison to CSF samples of living patients with probable CJD when analyzed by second generation RT-QuIC^15^.

In the presented study, we assessed the performance of second generation RT-QuIC assay in detection of prions in *post-mortem* skin and CSF samples of neuropathologically confirmed TSE and control non-TSE patients. We report proclivity of undiluted *post-mortem* CSF samples to inhibit RT-QuIC reactions and predisposition of some control skin samples to initiate non-specific PrP aggregation. Despite these complications our data confirm high diagnostic potential of CSF and skin sample RT-QuIC analysis.

## Results

### Patient cohorts

The skin and CSF samples were available from patients with definitive TSE (n = 38) and control patients with other autopsy confirmed diagnosis (n = 30). The post-mortem interval before autopsy was in the range from 6.5 to 146 h (Table S1). Majority of TSE patients were classified as sporadic CJD (n = 34), mainly of MM1 type (n = 16). Other CJD types were represented in lower numbers (n = 2–4) and included one case of rare variably protease-sensitive prionopathy (VPSPr). The genetic TSE cases included gCJD with E200K mutation (n = 2) and Gerstmann-Sträussler-Scheinker syndrome with P102L mutation (GSS, n = 2). Control group consisted mainly of patients with other neurodegenerative diagnosis dominated by Alzheimer disease (n = 12) and frontotemporal lobar degeneration (n = 8). Summary of demographic data and patients’ diagnosis in both experimental cohorts are in Table 1.

**Table 1:** Characteristics of the analyzed patient cohorts.

	n	Mean age ± SD	Male (n)/female (n)		n	Mean age ± SD	Male (n)/female (n)
**Sporadic TSEs**	**34**	**69 ± 10**	**18/16**	**Non-TSEs**	**30**	**72 ± 14**	**18/12**
CJD MM1	16	75 ± 8	12/4	AD	12	79 ± 8	8/4
CJD MM2	2	56, 59	0/2	DLB	2	89, 78	2/0
CJD VV1	3	59 ± 10	1/2	FTLD^a^	8	74 ± 6	5/3
CJD VV2	4	68 ± 11	2/2	VaD	1	94	0/1
CJD MV1	3	70 ± 4	1/2	Syn	1	56	1/0
CJD MV2	3	57 ± 12	1/2	ND-A	1	56	1/0
CJD MM1 + 2	2	63, 70	0/2	Lymphoma infiltration	1	54	0/1
CJD VPSPr	1	73	1/0	H/ABI	3	43 ± 8	1/2
**Genetic TSEs**	**4**	**62 ± 16**	**1/3**	Encephalitis, DLBCL	1	66	0/1
gCJD E200K	2	65, 75	1/1				
GSS P102L	2	38, 69	0/2				

The diagnosis of patients in both experimental groups was confirmed by neuropathological evaluation of brain tissue at autopsy.

*CJD* Creutzfeldt-Jakob disease, *VPSPr* variably protease-sensitive prionopathy, *gCJD* genetic CJD, *GSS* Gerstmann-Sträussler-Scheinker syndrome, *AD* Alzheimer disease, *DLB* dementia with Lewy’s bodies, *FTLD* frontotemporal lobar degeneration, *VaD* vascular dementia, *Syn* synucleinopathy, *ND-A* non-dementia-alcoholism, *H/ABI* hypoxic/anoxic brain injury, *DLBCL* diffuse large B-cell lymphoma.

^a^FTLD includes FTLD-UPS, FTLD-tau, FTLD-TDP, FTLD-U + TDP-43 and progressive supranuclear palsy.

### Detection of prion seeding activity in the CSF samples

**Figure 1:**
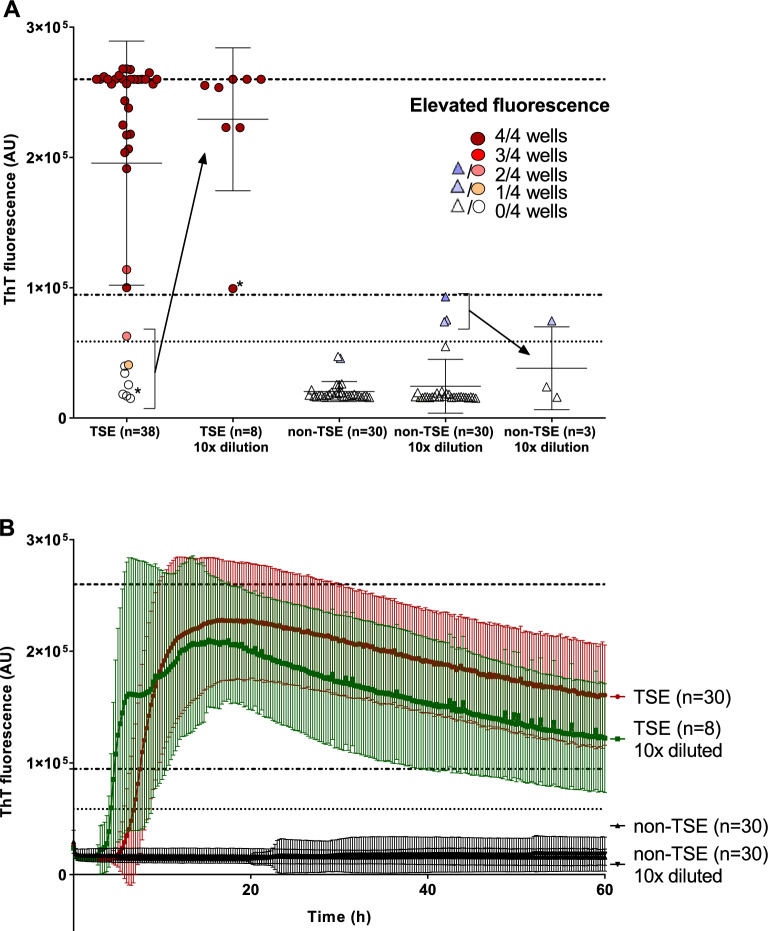
RT-QuIC detection of prion templating activity in *post-mortem* CSF samples. (**A**) Dot plot of mean max ThT fluorescence intensity of the CSF samples depicting mean ± SD. The samples were analyzed in quadruplicates and considered as positive when the mean fluorescence signal exceeded the threshold value (dotted line) and at least two wells showed elevated signal. Number of the wells with elevated signal in the quadruplicate is represented by the color intensity. Out of the 38 undiluted TSE CSF samples 8 gave low fluorescence signal, but they all became positive after the dilution to decrease the CSF protein content. The dash-dotted line represents the threshold value for diluted samples. No non-TSE control sample was positive (triangles). The three 10× diluted control samples with higher signal gave lower signal after reanalysis. Dashed line represents upper limit of the fluorescence measurement. (**B**) Time course of the mean of ThT fluorescence of undiluted TSE CSF samples (n = 30, red), diluted TSE CSF samples (n = 8, green) and non-TSE control samples (n = 30, black). The points and error bars represent mean fluorescence ± SD. *AU* arbitrary fluorescence units. *Variably protease-sensitive prionopathy (VPSPr).

The samples were analyzed by previously published protocol for *intra-vitam* CSF^8^. Out of 38 undiluted *post-mortem* TSE CSF samples 7 gave false-negative results and 1 was only borderline positive suggesting presence of aggregation inhibitors and the sensitivity of the assay 81.6%. However, all these 8 samples became positive after 10× dilution with PBS (Fig. 1A). The calculated fluorescence threshold value for 10× diluted CSF samples was higher than for undiluted CSF due to the presence of moderately increased signal of 3 non-TSE samples (Fig. 1A). None of the control CSF samples was assessed positive suggesting overall sensitivity and specificity of the assay 100%. The mean max ThT fluorescence of undiluted (n = 30) and diluted (n = 8) TSE samples were 23 ± 5 × 10^4^ and 21 ± 6 × 10^4^ AU, respectively, with number of the samples reaching the upper fluorescence limit of the assay (Fig. 1A). The corresponding time to threshold was 7.0 ± 5.3 h and 5.4 ± 4.1 h, respectively (Fig. 1B). Diluted CSF sample of VPSPr and one undiluted MV1 and E200K sample provided notably lower signal in comparison to the signals of other studied sporadic and genetic TSEs (Fig. S4). Mean ThT maximum response of undiluted control CSF samples 19 ± 5 × 10^3^ AU was significantly lower (*P* < 0.05) and safely below the threshold (Fig. 1B). In comparison, 3 diluted control samples (AD and 2 FTLD) provided higher signal, but still under the calculated threshold and their signal lowered after the reanalysis (Fig. 1A). Total area under the curve for undiluted (n = 30) and diluted (n = 8) TSE samples was 9.6 × 10^6^ and 8.4 × 10^6^, respectively, two orders of magnitude higher than 7.6 × 10^4^ and 1.4 × 10^5^ of the control undiluted and diluted samples (n = 30). The values of maximal ThT fluorescence of all analyzed CSF samples are presented in Table S1.

The protein content of CSF samples was 4.27 ± 2.27 and 5.38 ± 4.57 mg/ml in TSE and non-TSE group, respectively (Table S1). The range of the concentrations was between 1.37 and 9.70 mg/ml excluding one outlier (Fig. S11). The protein concentration of the undiluted CSF TSE samples producing low signal (n = 8) was higher (5.69 ± 2.50 mg/ml) than of the rest of TSE samples (3.39 ± 2.09 mg/ml), but the difference was not significant (P = 0.082). The length of post-mortem interval (PMI) moderately positively correlated with protein concentration of collected CSF samples (R = 0.49, p = 0.00003, Table S1). The PMI > 48 h led to twofold higher CSF protein concentration than PMI < 24 h (supplementary results). Median PMI (55 h) of the CSF samples producing low signal undiluted was significantly higher than that of the rest of TSE samples (29.5 h, p = 0.012).

### Detection of prion seeding activity in the skin using RT-QuIC protocol for CSF samples

First, we attempted to analyze skin samples using the same protocol as for the CSF samples^16^. We reasoned that utilization of large volume (15 µl) of skin homogenate may increase sensitivity of the assay. We have obtained positive signal in most TSE samples, but we also obtained equally high signal in six control non-TSE samples (Fig. S1). We repeated the analysis of non-TSE samples (n = 30) with freshly prepared skin homogenates and obtained similar number of false-positive reactions (Fig. S1). The attempt to suppress non-specific positive reactions by inclusion of N-2 supplement into the reaction mixture led to increase in the separation of the signal of positive and negative samples (Fig. S2), but many control samples still generated fluorescence signal above the baseline preventing the calculation of useful fluorescence threshold for distinguishing the positive and negative reactions. For more details see supplementary data.

### Detection of prion seeding activity in the skin using RT-QuIC protocol for brain samples

**Figure 2:**
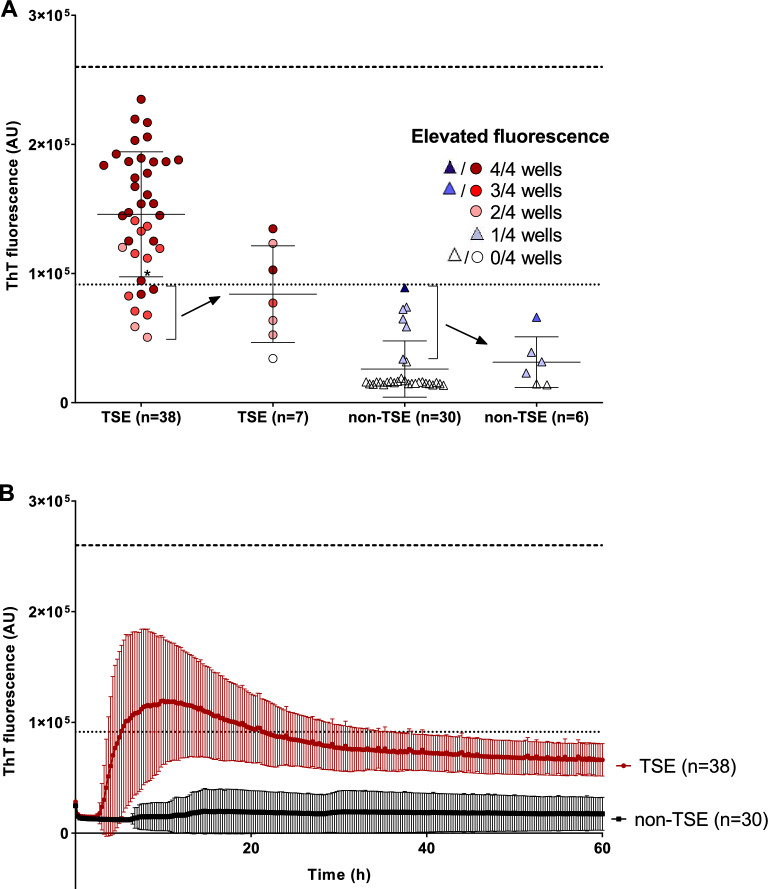
RT-QuIC detection of prion templating activity in *post-mortem* skin samples. (**A**) Dot plot of mean max ThT fluorescence intensity of the skin samples depicting mean ± SD. The samples were analyzed in quadruplicates and considered as positive when the mean fluorescence signal exceeded the threshold value (dotted line) and at least two wells showed elevated signal. Number of the wells with elevated signal in the quadruplicate is represented by the color intensity. Out of the 38 TSE skin samples (circles) seven did not reach the threshold and were reanalyzed (arrow). No non-TSE control skin sample was positive (n = 30, triangles). The control samples with the signal above the mean of the control group (n = 7) were reanalyzed and gave lower signal (arrow). Dashed line represents upper limit of the fluorescence measurement. (**B**) Time course of the mean ThT fluorescence of TSE skin samples (n = 38, red) and non-TSE controls (n = 30, black). The points and error bars represent mean fluorescence ± SD. *AU* arbitrary fluorescence units. *Variably protease-sensitive prionopathy (VPSPr).

Employment of the protocol for brain samples^16^ utilizing just 2 µl of 10 times diluted skin homogenate led to suppression of false-positive aggregations while preserving specific signal of most TSE samples. Out of 38 *post-mortem* TSE skin samples, 31 were classified as positive corresponding to 81.6% assay sensitivity (Fig. 2A). Seven TSE skin samples, including MM1 (n = 4), MV1 (n = 1), VV1 (n = 1) and GSS (n = 1), did not reach the fluorescence threshold (dotted line) even though they all demonstrated noticeable ThT fluorescence signal in two or more wells justifying their reanalysis. The VPSPr sample was just borderline positive while skin homogenates of MV2 (n = 3) and E200K (n = 2) patients tended to produce higher signals (Fig. S8). The mean max ThT fluorescence of positive TSE skin samples was 12 ± 5.7 × 10^4^ AU and the mean time to threshold was 5.5 ± 4.8 h. The non-TSE controls had significantly lower (sixfold) mean max ThT fluorescence 20 ± 21 × 10^3^ AU (*P* < 0.05), but the relative difference was smaller than between TSE and non-TSE CSF samples (12-fold). Total area under the curve for skin TSE samples was 3.9 × 10^6^ while for non-TSE control samples it was 2.7 × 10^5^ (Fig. 2B). Out of 7 TSE skin samples with insufficient ThT signal 3 became positive in repeated experiment improving the assay sensitivity to 89.5% (Fig. 2A). The signal of all non-TSE samples was below the calculated threshold, suggesting the specificity of the assay 100%, albeit 5 samples (FTLD-UPS, DLB, Syn, AD and H/ABI) showed fluorescence signal higher than the rest of controls. Out of them 4 gave the signal only in one well and their mean ThT fluorescence decreased in repeated experiment (Fig. 2A). Interestingly, the H/ABI sample manifested low, but evident ThT signal in all four wells and in three wells after the repetition. Control RT-QuIC of the corresponding brain homogenates of these 5 non-TSE patients was negative confirming the absence of TSE comorbidity not detected during the autopsy (not shown). The individual max ThT values of all analyzed skin samples are presented in Table S1.

Mean protein concentration of collagenase treated skin sample supernatants used to seed the RT-QuIC reactions was 4.40 ± 0.70 and 4.36 ± 1.36 mg/ml in TSE and non-TSE group, respectively (Table S1). The lowest and the highest recorded concentrations were 2.05 and 7.97 mg/ml (Fig. S12). Electrophoretic protein profile of the representative TSE and non-TSE samples after gel silver staining was comparable (Fig. S13). The protein concentration of TSE skin samples which gave negative reaction (4.08 ± 0.60 mg/ml; n = 7) was similar to the TSE samples which were RT-QuIC positive (4.48 ± 0.71 mg/ml).

### Estimation of median prion RT-QuIC seeding dose present in patients’ CSF and skin

The median seeding dose (SD_50_) was evaluated by limiting tenfold dilutions of representative mixed tissue samples of MM1 (n = 16), VV2 (n = 4), MV1 (n = 3) and MV2 (n = 3) CJD patients and of individual VPSPr sample. The Spearman-Karber analysis^17^ of data was applied to calculate SD_50_ (Table 2). The SD_50_ of mixed CSF samples varied between 10^5.6^/ml for MV2 and 10^7.1^/ml for VV2 with VPSPr CSF having SD_50_ 10^5.8^/ml. The SD_50_ of corresponding mixed skin samples was approximately one order of magnitude higher (*P* ˂ 0.05), between 10^6.9^/g for MV1 and 10^7.7^/g for MM1 with VPSPr at 10^7.7^/g. The original data of limiting dilution experiment are presented in supplemental Tables S2 and S3.

**Table 2:** Median seeding dose (SD_50_) of representative CSF and skin samples of CJD patients.

sCJD type	n	log_10_ SD_50_/15 µl CSF	log_10_ SD_50_/ml CSF	log_10_ SD_50_/2 µl skin homogenate	log_10_ SD_50_/g skin
MM1	16	4.75	6.6	5	7.7
VV2	4	5.25	7.1	4.75	7.4
MV1	3	4	5.8	4.25	6.9
MV2	3	3.75	5.6	4.75	7.4
VPSPr	1	4	5.8	5	7.7

Equal aliquots of individual patient samples were mixed to provide representative sample for different CJD types. The mixed samples were serially tenfold diluted and analyzed using RT-QuIC. The SD_50_ signifies dilution of the sample at which only half of the assay wells provides positive readout. *VPSPr* variably protease-sensitive prionopathy.

## Discussion

High value of CSF RT-QuIC analysis in living TSE patients was demonstrated in numerous studies and is already utilized to improve diagnosis of probable CJD in many developed countries. Studies utilizing *post-mortem* CSF or skin are comparatively sparse. Our study was carried out on the samples of both tissues collected concurrently at autopsy allowing unbiased comparison of the achieved results. The composition of *post-mortem* CSF considerably differs from *intra-vitam* CSF with the protein concentration being typically 10 times higher^18^. Recently, the inhibition of RT-QuIC by undiluted *post-mortem* CSF was reported and we have demonstrated previously that protein content in the *post-mortem* CSF over 2.5 mg/ml correlated with the inhibition of RT-QuIC reactions^16,19^. In our present study the CSF TSE samples inhibiting RT-QuIC reaction had significantly longer PMI and their mean protein concentration was 1.7 times higher than that of the samples giving positive results. However, on the individual level, two CSF samples which inhibited RT-QuIC reaction had relatively low protein content (< 2.5 mg/ml) and conversely two CSF samples with the highest protein content (~ 10 mg/ml) gave positive RT-QuIC results. It suggests that the inhibition of RT-QuIC by *post-mortem* CSF is patient specific and depends not only on the protein concentration, but also on the composition of individual CSF samples. Similar observation was made in recent study analyzing the nature of CSF inhibitors of alpha synuclein RT-QuIC^20^. What components of *post-mortem* CSF are responsible for prion RT-QuIC inhibition is not clear. The inhibitory effect of various substances including hemoglobin, mucin and brain phospholipids was reported already^21^. Previously simple dilution of the CSF was shown to prevent false negative results^16,22^ and this was confirmed also in our current study. Based on this experience it seems sensible to analyze *post-mortem* CSF samples simultaneously undiluted and tenfold diluted. The dilution of control non-TSE CSF samples led to detection of increased fluorescence signal in 3 control samples. While the signal of 2 of them lowered after the reanalysis, the signal of one sample remained higher. The inclusion of all diluted control samples resulted in calculation of higher fluorescence threshold. Higher fluorescence threshold for diluted CSF samples was noted also in our previous study^16^. The reason for this increase is not clear, but calculation of separate threshold for diluted CSF samples should decrease the chance of obtaining false positive result as was demonstrated also by our current data. Published studies differ in the ways of threshold estimation and it is not clear what approach is soundest. We have calculated the threshold as the mean maximal signal of all negative samples plus five standard deviations. Such threshold, in the case of normal data distribution, corresponds to just one false positive out of million negative samples. However, as the data failed normality test the likelihood of false positivity at this threshold is presumably higher and considerably larger control group would be needed for the odds precise estimation. Interestingly, the mean time to threshold of undiluted CSF samples was about 30% longer then for the diluted samples probably due to inhibitory effect of their high protein concentration. Despite this the second-generation RT-QuIC with shortened rHaPrP90-231 substrate provided positive results for *post-mortem* CSF of all tested TSE subtypes including VPSPr in less than 20 h confirming the ability of the assay to deliver diagnostic information within one day. Our data verified excellent potential of *post-mortem* CSF samples for RT-QuIC confirmation of TSE diagnosis. Sampling of CSF from lateral ventricle after the skull opening is relatively straightforward procedure and CSF requires minimal handling before the RT-QuIC analysis. This makes *post-mortem* CSF an attractive alternative to sample of brain tissue.

The analysis of the corresponding *post-mortem* skin samples was just slightly more complex despite the anticipated higher natural variation in the composition of skin samples of individual patients. Previous studies have tested utilization of skin samples from different body areas and demonstrated variable outcomes^12,14^. Among the areas providing most consistent results were neck and the area behind the ear which was utilized also in our current study. For preparation of the skin lysates we have exploited method described by Orru et al*.*^12^ based on collagenase A digestion and sonication. Our preliminary experiments have confirmed the presence of prion templating activity in the clarified skin lysates of TSE patients. We also noted the absence of obvious lysate RT-QuIC inhibiting activity which led us to decision to analyze the samples just minimally diluted. We have analyzed the CSF and skin samples on the same plate reader and with identical fluorescence settings to be able to directly compare the obtained RT-QuIC data. The fluorescence signal of skin samples was notable lower than with the corresponding CSF and in contrast to many CSF samples never reached the upper fluorescence limit of the assay. At the other hand, the time to threshold of skin samples was slightly shorter than of undiluted CSF samples. The reason for this disparity is not clear and illustrates the complex effects of the sample matrix on the RT-QuIC data. Occasionally, we noted inclination of the non-TSE control skin lysates to produce elevated RT-QuIC signal. This was more pronounced when volume of the lysate used to seed RT-QuIC was higher. Decreasing of the seeding volume and inclusion of N-2 supplement improved the separation of TSE and non-TSE control results. Even though our study was not blinded the estimated achieved sensitivity 89.5% using second-generation RT-QuIC was similar to the sensitivity reported by Mammana et al. and Orru et al*.* utilizing bank vole rPrP as RT-QuIC substrate^12,14^. The fact that studies differing in the skin homogenization procedure, recombinant substrate sequence and the RT-QuIC conditions gave comparable data is highly encouraging for further development of diagnostic use of the skin samples. This is supported also by higher calculated SD_50_ of *post-mortem* skin mixed samples found in our end-point dilution analysis. The corresponding CSF samples had lower SD_50_ values irrespective of the sCJD type. Interestingly, recent study of Xiao et al*.* described notably higher sensitivity of skin RT-QuIC (91%) compared to just 45% sensitivity of CSF RT-QuIC in living Chinese patients with probable CJD^15^. Published study in animal model demonstrated that RT-QuIC is able to detect prions in the skin of infected animals before the development of TSE symptoms^13^. In our hands, the limitation of RT-QuIC skin analysis stems from the occasional propensity of TSE negative skin samples to generate false positive signal. This limitation may be of technical origin reflecting difficulties of skin sample preparation due to the differences in the skin composition of individual patients. Further studies are needed to clarify if the skin biopsy samples can serve as an alternative analyte to CSF and nasal brushings for confirmation of diagnosis in living TSE patients. Detection of prions in both, CSF and skin sample, could prove to be the way how to achieve definitive TSE diagnosis *intra-vitam*. Our study suggests that the employment of second-generation RT-QuIC assay with shortened hamster PrP substrate may allow simultaneous analysis of CSF and skin samples in one plate making the confirmation of TSE diagnosis simpler.

## Methods

### Patients

The samples were provided by the Czech National Reference Laboratory for Human Prion Diseases. The study was approved by ethical committee of the Institute for Clinical and Experimental Medicine and Thomayer University Hospital in Prague (G-16-06-39). The ethical committee of the Institute for Clinical and Experimental Medicine and Thomayer University Hospital in Prague waived the need for informed consent due to the study's retrospective nature. The research was performed following the Declaration of Helsinki. A total of 68 pairs of skin and CSF *post-mortem* samples were available from patients with neuropathologically confirmed definitive TSE (n = 38) and from controls with other neurodegenerative (n = 24) or non-neurodegenerative (n = 6) diseases, where TSE were neuropathologically excluded. The characteristics of the experimental cohorts are provided in Table 1.

### Preparation of human skin and CSF samples

The skin samples were taken at autopsy from the area behind the ear using sterile scalpel before the skull opening to prevent possibility of cross contamination. The CSF was aspirated using sterile needle from lateral ventricle. Care was taken to avoid CSF contamination with the brain tissue. Both tissues were immediately frozen at – 80 °C.

The CSF samples were thawed and centrifuged for 2 min at 2000*g* before adding the supernatant into the reaction mix. Eventually, the CSF was 10× diluted with PBS (pH 7.4).

The skin sample homogenates were prepared as described previously^12^. Briefly, a small piece (30–60 mg) containing all three skin layers was washed in ice cold PBS. The 10% homogenate was prepared by incubating the sample with 0.25% collagenase A (no. 10103586001, Roche) in TBS, 2 mM CaCl_2_ (pH 7.4) at 37°C and 300 rpm for 4 h (Thermomixer R, Eppendorf). Subsequently, the samples were sonicated for 30 s (Cup Horn probe, CPX750 sonicator, Cole-Parmer) and centrifuged at 500*g* for 7 min. The pellet was discarded, and the supernatant centrifuged again at 2000*g* for 2 min. The supernatant was 10× diluted in PBS containing 1× N-2 supplement (no. 17502048, Gibco) and 0.1% SDS before adding to the reaction mix.

The protein content of the CSF samples and skin lysate supernatants was measured by Pierce BCA protein assay (Thermo Fisher Scientific). The protein pattern of the skin samples was analyzed by SDS PAGE and silver staining of the gels as described previously^23^.

### Purification of the recombinant prion protein

Syrian hamster recombinant prion protein (rHaPrP90-231) was prepared according to published protocol^8^. Shortly, inclusion bodies produced by *E. coli* were solubilized in 8 M guanidine HCl and the protein purified using chromatography on Ni-Sepharose™ 6 Fast Flow (Cytiva). The rHaPrP90-231 was refolded on the column, eluted with a gradient of imidazole and dialyzed^16^. The protein absorbance was measured and 0.22 μm filtered aliquots stored at – 80 °C.

### Detection of prion seeding activity by RT-QuIC

The samples were analyzed by second-generation RT-QuIC assay^5,24^. Reaction mixture (98 or 85 µl) composed of 11.9 mM phosphate, 1 mM EDTA, 310 mM NaCl (pH 7.4), 10 µM Thioflavin T, 0.1 mg/ml rHaPrP90-231 and 0.002% SDS (for CSF samples) was loaded into the wells of 96-well plate and seeded with 2 µl of skin homogenate or 15 µl of CSF, respectively. The plate was incubated at 55°C in FLUOstar OMEGA reader (BMG) with repeating 1 min cycles of shaking at 700 rpm and 1 min rest for 60 h. Fluorescence signal was taken every 15 min. The sample was classified as positive if the mean maximal ThT fluorescence of four replicates was higher than the threshold and at least two wells showed elevated signal above the fluorescence baseline. If the elevated signal was detected only in one well the assay was repeated. The assay was repeated also if the signal was present in two or more wells but it did not exceeded the threshold. The sample was classified positive if at least two wells out of 8 replicates showed the signal and the mean maximal ThT fluorescence of the replicates was above the threshold. The threshold was calculated as the average of maximal ThT fluorescence of individual control non-TSE samples plus 5 standard deviations (SD). In the case of control skin samples the 6 samples which provided detectable fluorescence signal (Fig. 2A) were reanalyzed and the mean fluorescence of all 8 replicates was included in the skin threshold calculation. The threshold for undiluted and diluted CSF samples was calculated separately and the mean fluorescence of all 8 replicates of 3 reanalyzed diluted CSF samples (Fig. 1A) was used in the calculation.

### Estimation of median prion RT-QuIC seeding dose (SD_50_) of CSF and skin samples

The identical aliquots of CSF or skin homogenate of individual patients within the same CJD type group were pooled to provide representative samples for limiting dilution experiment. The samples were tenfold serially diluted and analyzed by RT-QuIC. The SD_50_ was calculated using Spearman-Karber analysis^17^_._

### Statistical analysis

The results were plotted using GraphPad Prism 5 (GraphPad Software Inc.) and statistically analyzed using Sigma Stat nonparametric Mann–Whitney U test (Systat Software Inc.). The difference was assumed significant for *P* value ˂ 0.05.

### Supplementary Information

Supplementary Information.

## Data Availability

The datasets analyzed in the current study are available from the corresponding author on reasonable request.

## References

[1] Collinge J (2016). Mammalian prions and their wider relevance in neurodegenerative diseases. Nature.

[2] Kang HE, Mo Y, Abd Rahim R, Lee HM, Ryou C (2017). Prion diagnosis: Application of real-time quaking-induced conversion. Biomed. Res. Int..

[3] Collins SJ (2006). Determinants of diagnostic investigation sensitivities across the clinical spectrum of sporadic Creutzfeldt-Jakob disease. Brain.

[4] Cramm M (2016). Stability and reproducibility underscore utility of RT-QuIC for diagnosis of Creutzfeldt-Jakob disease. Mol. Neurobiol..

[5] Wang F, Pritzkow S, Soto C (2023). PMCA for ultrasensitive detection of prions and to study disease biology. Cell Tissue Res..

[6] Takatsuki H (2015). Rapid and quantitative assay of amyloid-seeding activity in human brains affected with prion diseases. PLoS ONE.

[7] Atarashi R (2011). Ultrasensitive human prion detection in cerebrospinal fluid by real-time quaking-induced conversion. Nat. Med..

[8] Orru CD (2015). Rapid and sensitive RT-QuIC detection of human Creutzfeldt-Jakob disease using cerebrospinal fluid. mBio.

[9] Orrú CD (2020). Ring trial of 2nd generation RT-QuIC diagnostic tests for sporadic CJD. Ann. Clin. Transl. Neurol..

[10] Orrú CD (2014). A test for Creutzfeldt-Jakob disease using nasal brushings. N. Engl. J. Med..

[11] Bongianni M (2017). Diagnosis of human prion disease using real-time quaking-induced conversion testing of olfactory mucosa and cerebrospinal fluid samples. JAMA Neurol..

[12] Orru CD (2017). Prion seeding activity and infectivity in skin samples from patients with sporadic Creutzfeldt-Jakob disease. Sci. Transl. Med..

[13] Wang Z (2019). Early preclinical detection of prions in the skin of prion-infected animals. Nat. Commun..

[14] Mammana A (2020). Detection of prions in skin punch biopsies of Creutzfeldt-Jakob disease patients. Ann. Clin. Transl. Neurol..

[15] Xiao K (2021). Validation and application of skin RT-QuIC to patients in China with probable CJD. Pathogens.

[16] Mosko T, Galuskova S, Matej R, Bruzova M, Holada K (2021). Detection of prions in brain homogenates and CSF samples using a second-generation RT-QuIC assay: A useful tool for retrospective analysis of archived samples. Pathogens.

[17] Wilham JM (2010). Rapid end-point quantitation of prion seeding activity with sensitivity comparable to bioassays. PLoS Pathog..

[18] Arroyo A, Rosel P, Marron T (2005). Cerebrospinal fluid: Postmortem biochemical study. J. Clin. Forensic Med..

[19] Mok TH (2021). Bank vole prion protein extends the use of RT-QuIC assays to detect prions in a range of inherited prion diseases. Sci. Rep..

[20] Bellomo G (2023). Cerebrospinal fluid lipoproteins inhibit alpha-synuclein aggregation by interacting with oligomeric species in seed amplification assays. Mol. Neurodegener..

[21] Hoover CE, Davenport KA, Henderson DM, Zabel MD, Hoover EA (2017). Endogenous brain lipids inhibit prion amyloid formation in vitro. J. Virol..

[22] Fiorini M (2020). High diagnostic accuracy of RT-QuIC assay in a prospective study of patients with suspected sCJD. Int. J. Mol. Sci..

[23] Brouckova A, Holada K (2009). Cellular prion protein in blood platelets associates with both lipid rafts and the cytoskeleton. Thromb. Haemost..

[24] Franceschini A (2017). High diagnostic value of second generation CSF RT-QuIC across the wide spectrum of CJD prions. Sci. Rep..

