# Supplementary material for: Detection of prions in matching post-mortem skin and cerebrospinal fluid samples using second-generation real-time quaking-induced conversion assay
Source: Sci Rep. 2024 Mar 15;14:6294. doi: 10.1038/s41598-024-56789-6 (PMC10943125; doi:10.1038/s41598-024-56789-6)
Supplement: Supplementary file 1 — Supplementary Information. [file 41598_2024_56789_MOESM1_ESM.pdf]

SUPPLEMENTARY MATERIAL

Detection of prions in matching *post-mortem* skin and cerebrospinal fluid samples using second-generation RT-QuIC assay

Soňa Galušková[1], Tibor Moško[1], Magdalena Brůžová[2], Tracy Haldiman[3], Chae Kim[3], Jiri G. Safar[3], Radoslav Matěj[2,4], and Karel Holada[1]*

[1] Institute of Immunology and Microbiology, First Faculty of Medicine, Charles University, Prague, Czech Republic.
[2] Department of Pathology and Molecular Medicine, Third Faculty of Medicine, Charles University and Thomayer University Hospital, Czech Republic.
[3] Departments of Pathology and Neurology, Case Western Reserve University School of Medicine, Cleveland, OH 44106, USA.
[4] Department of Pathology, Third Faculty of Medicine, Charles University and University Hospital Kralovske Vinohrady, Prague, Czech Republic.

Supplementary results and discussion

The length of *post-mortem* interval (PMI) affects protein concentration of ventricular CSF

The individual values of PMI and CSF protein concentration are presented in Table S1. All TSE (n=38) and non-TSE samples (n=30) were divided into 3 groups according to the length of their PMI and mean CSF protein concentration was calculated. Mean CSF protein concentration in Group A (PMI < 24h, n=23) was 3.53 ± 2.84 mg/ml; in Group B (PMI 24 – 48h, n=29) was 4.26 ± 1.93 mg/ml and in Group C (PMI > 48h, n=15) was 7.57 ± 5.18 mg/ml. The differences between the groups were statistically significant (P<0.01). Our results are in accord with previously published data demonstrating that protein concentration of CSF increases with the length of PMI[1].

Detection of prion seeding activity in the skin using RT-QuIC protocol for CSF samples

Initially, having in mind presumably low quantity of PrP^{Sc} in the skin, we have utilized the identical RT-QuIC protocol as was used for the CSF samples. We reasoned that seeding the RT-QuIC reaction with higher volume of the skin homogenate (15 µl into 85 µl of reaction mixture) may increase the sensitivity of the assay. The mean signal of TSE skin samples (n=38) was notably higher than the mean signal of non-TSE control skin samples (n=30) (Fig. S1A). However, a portion of the control samples (n=6) produced high false positive signal which overlapped with the signal of TSE samples (Fig. S1A, color circles). To assess if the ability to produce high false positive signal represents the nature of particular non-TSE samples we repeated their analysis utilizing newly prepared skin homogenates. The mean signal of the new control samples (n=30) was slightly higher and a portion of the samples (n=5) produced high false positive signal (Fig. S1A). However, out of them only two produced high signal in the previous experiment suggesting that the high signal of some non-TSE skin samples might be of technical origin e.g. caused by irregularities in the processing of the skin homogenates. At the same time two samples produced high signal in both skin homogenate preparations (Fig. S1A) and one of them (hypoxic/anoxic brain injury sample) was consistently producing higher

fluorescent signal in all four wells of the sample quadruplicate. The diagnosis of hypoxic/anoxic brain injury was confirmed by autopsy and RT-QuIC of the patient brain tissue was negative excluding the concomitant presence of TSE (not shown). The same sample produced repeatedly highest non-specific fluorescence also when the RT-QuIC protocol for brain tissue was utilized in the analysis of skin samples (main text, Fig. 2A). This suggests that on occasion some control non-TSE skin samples may have inherited propensity to cause false positive RT-QuIC signal.

Next we attempted to decrease the non-specific RT-QuIC signal of the control non-TSE samples by inclusion of N-2 supplement[2,3] into the buffer used for the skin homogenate dilution. N-2 supplement was used previously to improve detection of low levels of prion converting activity in highly diluted brain samples. Inclusion of N-2 supplement led to better separation of TSE and control results, still the fluorescence signal of non-TSE samples was significantly spread and did not allow establishment of useful fluorescence threshold (Fig. S2A). Insufficient separation of the TSE and non-TSE sample signals is documented also by the time course curves (Fig. S1B, Fig. S2B). The separation was greatly improved by the utilization of RT-QuIC protocol for brain samples which uses only 2 µl of the homogenate to seed the reaction (main text, Fig. 2B).

Taken together our data suggest that while high protein content of *post-mortem* CSF samples may often inhibit RT-QuIC reaction, the utilization of *post-mortem* skin collagenase homogenates might be connected with occasional tendency of the samples to generate false positive signal. However, both these obstacles can be solved either by simple CSF dilution or by using the RT-QuIC brain protocol with N-2 supplement for analysis of the skin samples.

References

1 Morris JA, Harrison LM, Telford DR. Postmortem cerebrospinal fluid pleocytosis: a marker of inflammation or postmortem artifact? Int J Pediatr. 2012;2012:964074. doi: 10.1155/2012/964074. Epub 2012 Feb 27. PMID: 22518189; PMCID: PMC3299252.

2 Bottenstein JE, Sato GH. Growth of a rat neuroblastoma cell line in serum-free supplemented medium. Proc Natl Acad Sci U S A. 1979 Jan;76(1):514-7. doi: 10.1073/pnas.76.1.514. PMID: 284369; PMCID: PMC382972.

3 Sünwoldt J, Bosche B, Meisel A, Mergenthaler P. Neuronal Culture Microenvironments Determine Preferences in Bioenergetic Pathway Use. Front Mol Neurosci. 2017 Sep 29;10:305. doi: 10.3389/fnmol.2017.00305. PMID: 29085280; PMCID: PMC5649214.

Supplementary tables:

TSE patient samples (n=38)									
**Diagnosis**	**Case no. / year of autopsy**	**PMI (hrs)**	**CSF max ThT FL (AU) x10^4**	**CSF 10x dil. max ThT FL (AU) x10^4**	**CSF 10x dil. reanalysis max ThT FL (AU) x10^4**	**CSF protein conc. (mg/ml)**	**Skin max ThT FL (AU) x10^4**	**Skin reanalysis max ThT FL (AU) x10^4**	**Skin protein conc. (mg/ml)**
**sCJD MM1** (n=16)	48/18	**26**	22			*4.1*	20		*3.7*
	**93/18**	**59**	22			*6.3*	**5.1**	**7.7**	*4.6*
	**121/18**	**42.5**	**6.2**	26*		*6.4*	14		*2.7*
	9/19	**24**	26*			*1.5*	14		*4.6*
	27/19	**29**	26*			*3.7*	18		*4.1*
	30/19	**35**	26*			*2.5*	15		*4.2*
	65/19	**40**	26*			*3.8*	19		*4.1*
	94/19	**16**	26*			*9.7*	17		*6.2*
	**100/19**	**46**	**3**	26*		*8.1*	15		*4.1*
	122/19	**79**	26*			*4.4*	15		*4.6*
	123/19	**35**	26*			*2.9*	16		*4.5*
	**16/20**	**30**	26*			*5.1*	**6.8**	**5.3**	*3.3*
	24/20	**34**	26*			*3.3*	11		*5*
	48/20	**15**	26*			*9.5*	14		*5.4*
	**50/20**	**22**	26*			*2.5*	**5.9**	**3.4**	*4.2*
	**66/20**	**96**	**3**	26*		*1.8*	**7.1**	**6.4**	*3.2*
**sCJD MM2** (n=2)	33/19	**30.5**	26*			*3*	12		*5*
	23/20	**36**	24			*3.8*	12		*5.1*
**sCJD VV1** (n=3)	**66/18**	**55**	**4**	22		*8.2*	**8.2**	12.3	*4.5*
	44/19	**14**	24			*1.4*	23		*3.3*
	56/19	**17**	26*			*3.3*	13		*3.8*
**sCJD VV2** (n=4)	63/19	**22**	26*			*2.9*	12		*4.7*
	**93/19**	**71**	**4**	22		*6.5*	19		*4.6*

	60/20	**38**	24			*2.9*	13		*5.7*
	67/20	**26**	26*			*6.2*	19		*3.8*
**sCJD MV1** (n=3)	**13/19**	**66**	**2**	25		*7.4*	19		*4.6*
	**130/19**	**16.5**	26*			*2.5*	**8.4**	13.5	*4.6*
	35/20	**36**	11			*6.9*	13		*4.1*
**sCJD MV2** (n=3)	30/18	**17**	21			*4.4*	19		*4.7*
	49/18	**39**	26*			*2.4*	22		*4.9*
	120/18	**11**	22			*2*	18		*5.1*
**sCJD MM1+2** (n=2)	75/19	**35**	20			*3.8*	22		*4.4*
	88/19	**17**	26*			*2*	14		*3.8*
**VPSPr** (n=1)	**111/18**	**8**	**2**	10		*2.3*	9.5		*4.6*
**gCJD E200K** (n=2)	85/19	**43**	10			*3.3*	20		*3.4*
	14/20	**47**	26*			*5*	19		*4.8*
**GSS P102L** (n=2)	**52/20**	**NA**	**2**	26*		*4.7*	**9**	10.3	*4.1*
	117/18	**6.5**	19			*1.5*	17		*4.6*

Control non-TSE samples (n=30)

**Diagnosis**	**Case no. / year of autopsy**	**PMI (hrs)**	**CSF max ThT FL (AU) x10^4**	**CSF 10x dil. max ThT FL (AU) x10^4**	**CSF 10x dil. reanalysis max ThT FL (AU) x10^4**	**CSF protein conc. (mg/ml)**	**Skin max ThT FL (AU) x10^4**	**Skin reanalysis max ThT FL (AU) x10^4**	**Skin protein conc. (mg/ml)**
**AD** (n=12)	61/18	**77**	1.9	1.9		*6.3*	1.4		*6.1*
	78/18	**22**	2.6	1.6		*11.6*	1.9		*2.6*
	84/18	**23**	1.7	1.6		*2.3*	1.4		*3.5*
	85/18	**140**	4.7	1.8		*22.7*	1.6		*5*
	87/18	**35**	1.6	1.5		*3.8*	1.5		*5.1*
	**94/18**	**46**	4.6	**9.3**	2.4	*6.4*	**5.9**	**3.9**	*5*
	102/18	**95.5**	1.7	1.7		*4.4*	1.6		*8*
	119/18	**34**	1.7	2.1		*2.8*	1.5		*4.6*

	129/18	**44**	1.6	1.7		*2.6*	1.5		*6.7*
	133/18	**22.5**	1.6	1.6		*1.8*	1.5		*4.4*
	140/18	**88**	1.7	1.9		*2.3*	1.6		*4.4*
	**16/19**	**12**	1.7	1.6		*2*	**3.4**	1.5	*5.3*
**FTLD** (n=8)	**80/18**	**37**	2	**7.5**	1.6	*4.7*	1.6		*4.6*
	12/19	**146**	1.9	5.5		*4.7*	1.5		*3.6*
	**36/19**	**80.5**	1.9	**7.4**	**7.5**	*12*	1.7		*6.1*
	40/19	**22**	1.7	1.6		*2.9*	1.5		*4.4*
	55/19	**95**	2.6	1.6		*10.5*	1.5		*4.9*
	62/19	**16**	1.7	1.9		*2.8*	1.3		*4.1*
	**96/19**	**24**	1.6	1.6		*5*	**7.4**	1.4	*5.4*
	128/19	**14**	1.7	1.6		*1.9*	1.6		*5.5*
**DLB** (n=2)	**71/19**	**42**	1.7	1.6		*1.4*	**7.2**	**3.2**	*3.9*
	78/19	**27**	1.8	1.6		*2.3*	1.4		*4*
**VaD** (n=1)	83/19	**30**	1.8	1.6		*5.4*	1.5		*2*
**Syn** (n=1)	11/19	**22**	1.7	1.6		*3.7*	**6.5**	**2.3**	*6.6*
**ND-A** (n=1)	74/19	**29.5**	1.7	1.6		*2.7*	1.7		*3.2*
**lymphoma infiltration** (n=1)	51/19	**34**	1.7	1.5		*4.2*	1.6		*3.2*
**H/ABI** (n=3)	**10/19**	**62**	1.7	1.6		*4.6*	**8.9**	**6.6**	*3.8*
	88/18	**68**	2.2	1.6		*11.5*	1.7		*4.1*
	**62/18**	**26**	2.6	1.6		*10*	3.2		*3.4*
**Encephalitis, DLBCL** (n=1)	71/18	**22**	1.7	1.6		*1.7*	1.6		*3.3*

*detection limit of the fluorescence reader

__Table S1:__ **Summarizing table showing time from the death to collection of samples at autopsy, maximal ThT fluorescence achieved in RT-QuIC experiments and protein concentration for each individual sample.** The maximal ThT fluorescence values of CSF samples are provided for undiluted CSF, 10x diluted CSF and for reanalyzed 10x diluted CSF. For skin samples the maximal ThT fluorescence values of 10x diluted 10% skin homogenates and for the identical reanalyzed samples are provided. The TSE samples which gave negative RT-QuIC in undiluted CSF (n=4) are in **bold blue**. The TSE samples with negative RT-QuIC in skin (n=4) are in **bold green** and the samples which were negative in both CSF and skin (n=3) are in **bold red**. Analogically, control non-TSE samples which provided higher RT-QuIC signal in diluted CSF (n=2) are in **bold blue**. The control samples with higher signal in the skin (n=5) are in **bold green**, and one non-TSE sample which provided higher signal in both, skin and diluted CSF is in **bold red**. The calculated fluorescence threshold values were 5.87 x 10^4 AU for undiluted CSF samples, 9.46 x 10^4 AU for 10x diluted CSF samples and 9.15 x 10^4 AU for skin samples.

PMI - *post-mortem* interval in hours (bold), CSF – cerebrospinal fluid, CSF 10x dil. – cerebrospinal fluid 10-fold diluted with PBS, max ThT FL - maximal ThT fluorescence, AU - arbitrary fluorescence units. Protein concentration of undiluted CSF and 10% skin homogenate is in *italics* (mg/ml). CJD – Creutzfeldt-Jakob disease, VPSPr – variably protease-sensitive prionopathy, gCJD – genetic CJD, GSS – Gerstmann-Sträussler-Scheinker syndrome, AD – Alzheimer disease, DLB – dementia with Lewy´s bodies, FTLD – frontotemporal lobar degeneration, VaD – vascular dementia, Syn – synucleinopathy, ND-A – non-dementia-alcoholism, H/ABI – hypoxic/anoxic brain injury, DLBCL – diffuse large B-cell lymphoma.
*FTLD includes FTLD-UPS, FTLD-tau, FTLD-TDP, FTLD-U+TDP-43 and progressive supranuclear palsy.

sCJD type	no. of replicates	no. of positives at 10^{-3}	no. of positives at 10^{-4}	no. of positives at 10^{-5}	no. of positives at 10^{-6}
**MM1** (n=16)	4	4	3	2	0
**VV2** (n=4)	4	4	4	3	0
**MV1** (n=3)	4	4	2	0	0
**MV2** (n=3)	4	4	1	0	0
**VPSPr** (n=1)	4	4	4	1	0

Table S2: Estimation of median seeding dose (SD_{50}) present in CSF samples. Equal aliquots of individual patient samples were mixed to provide representative sample for different CJD types. The mixed samples were serially 10-fold diluted and analyzed in quadruplicate using RT-QuIC. Number of replicates giving positive reaction at given CSF dilution is presented. The values of calculated SD_{50} are shown in Table 2.

sCJD type	no. of replicates	no. of positives at 10^{-3}	no. of positives at 10^{-4}	no. of positives at 10^{-5}	no. of positives at 10^{-6}
**MM1** (n=16)	4	4	4	2	0
**VV2** (n=4)	4	4	4	1	0
**MV1** (n=3)	4	4	2	1	0
**MV2** (n=3)	4	4	4	1	0
**VPSPr** (n=1)	4	4	4	2	0

Table S3: Estimation of median seeding dose (SD_{50}) present in skin samples. Equal aliquots of individual patient skin 10% homogenates were mixed to provide representative sample for different CJD types. The mixed samples were serially 10-fold diluted and analyzed in quadruplicate using RT-QuIC. Number of replicates giving positive reaction at given skin homogenate dilution is presented. The calculated values of SD_{50} are presented in Table 2.

Supplementary figures:

Figure S1

A)

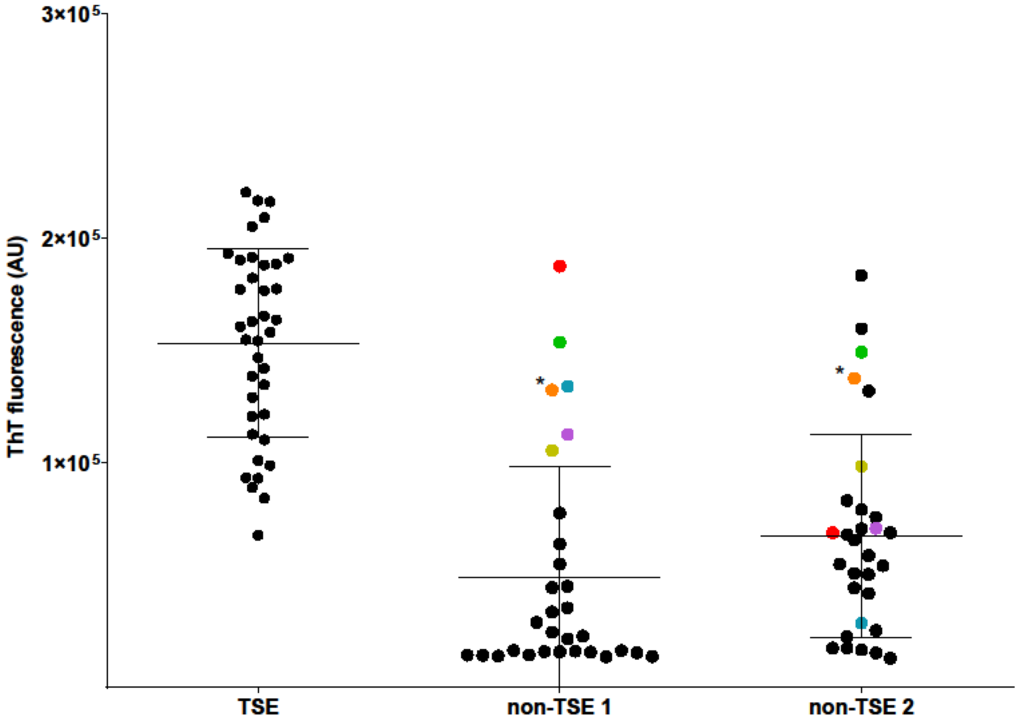

B)

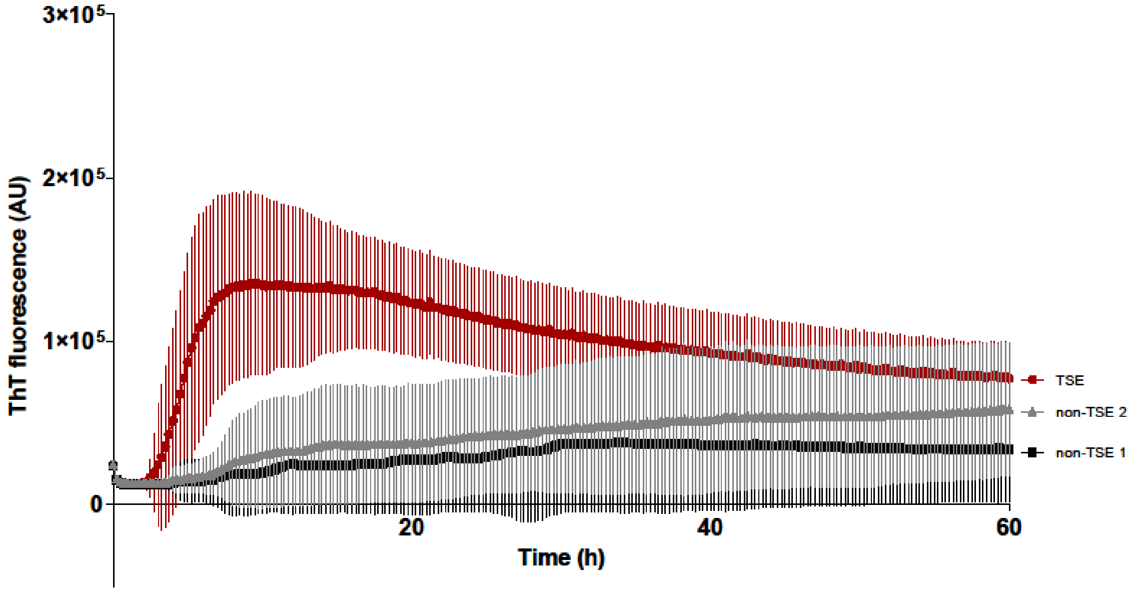

RT-QuIC detection of prion templating activity in frozen *post-mortem* skin samples using protocol for CSF samples. (A) Dot plot of mean max ThT fluorescence intensity of the skin samples analyzed in quadruplicates depicting mean ± SD. The mean signal of TSE skin samples (n=38) was notably higher than the mean signal of control skin samples (non-TSE 1, n=30). Portion of the control samples (n=6) produced high false positive signal (color circles). To check if the false positive signal is reproducible, the assay was repeated with newly prepared skin homogenates (non-TSE 2, n=30). The position of

the false positive samples from the first experiment is demonstrated (color circles). Asterisk labels hypoxic/anoxic brain injury skin sample. (B) Time course of the mean ThT fluorescence of TSE skin samples (n=38, red), non-TSE 1 controls (n=30, black) and non-TSE 2 controls (n=30, gray). The points and error bars represent mean fluorescence ± SD. AU - arbitrary fluorescence units.

<u>Figure S2</u>

A)

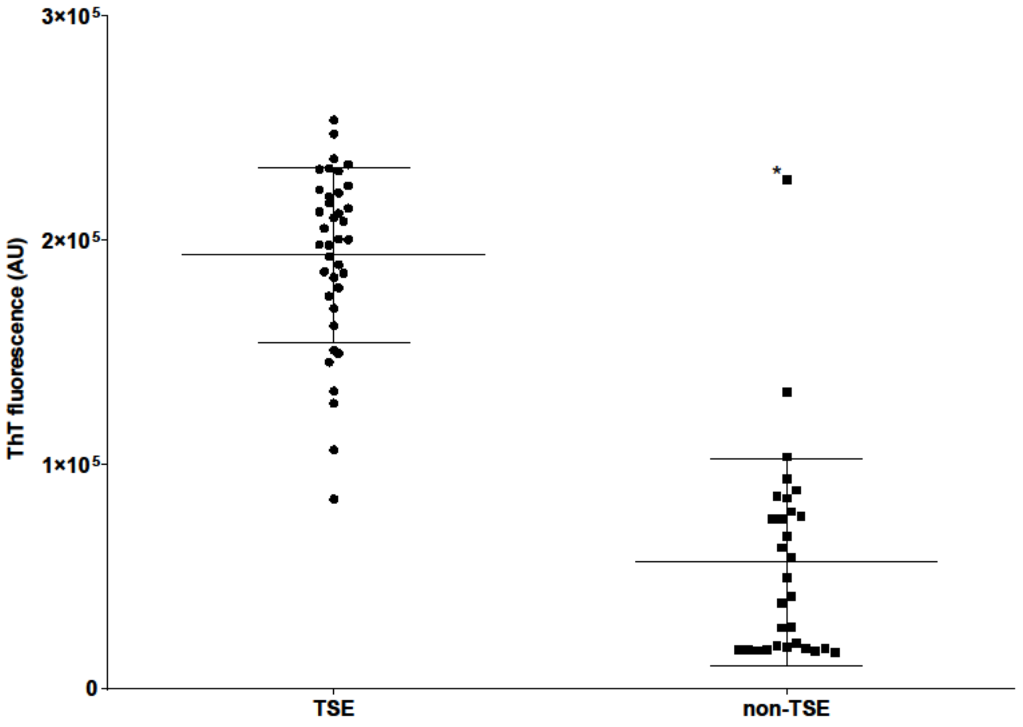

B)

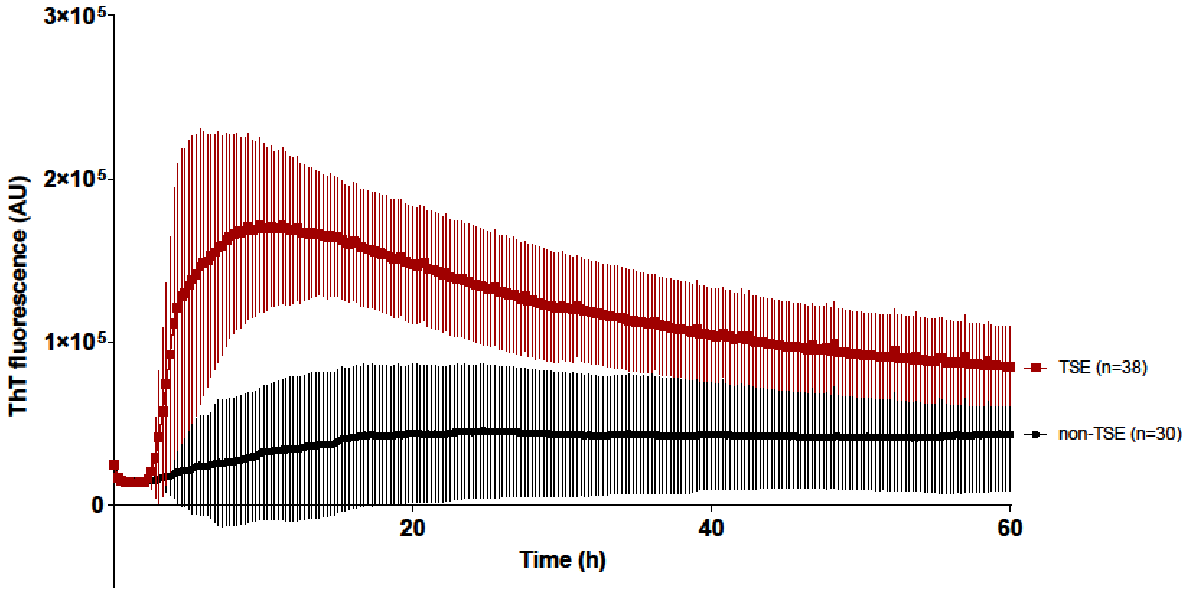

The effect of N-2 supplement on detection of prion templating activity in frozen *post-mortem* skin samples using RT-QuIC protocol for CSF samples. (A) Dot plot of mean max ThT fluorescence intensity of the skin samples analyzed in quadruplicates depicting mean ± SD. The mean signal of TSE

skin samples (n=38, circles) was notably higher than the mean signal of non-TSE control skin samples (n=30, squares). The high spread of the fluorescence signal of the control samples prevented the establishment of useful fluorescence threshold. Asterisk labels hypoxic/anoxic brain injury skin sample. (B) Time course of the mean ThT fluorescence of TSE skin samples (n=38, red) and non-TSE controls (n=30, black). The points and error bars represent mean fluorescence ± SD. AU - arbitrary fluorescence units.

<u>**Figure S3**</u>

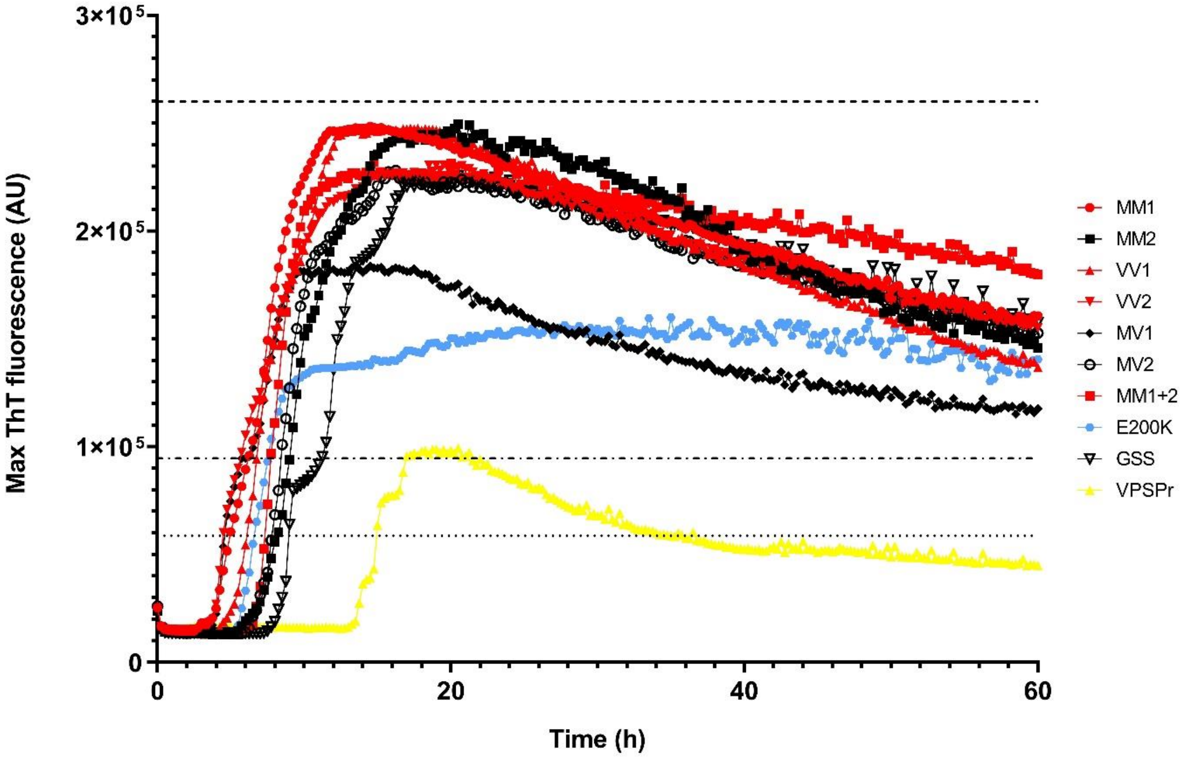

RT-QuIC time course of the mean ThT fluorescence of TSE *post-mortem* CSF samples grouped according to the disease type. The size of the groups varied from n = 16 for CJD MM1 to n = 1 for VPSPr and the description of groups is present in Table 1. The traces were calculated from the signal of undiluted CSF samples (n=30) and diluted CSF samples (n=8) for the samples which were negative undiluted. The points represent mean fluorescence. The dotted line and dash-dotted line represent the threshold value for undiluted and diluted CSF samples, respectively. Dashed line depicts the upper limit of the fluorescence measurement. AU - arbitrary fluorescence units.

Figure S4

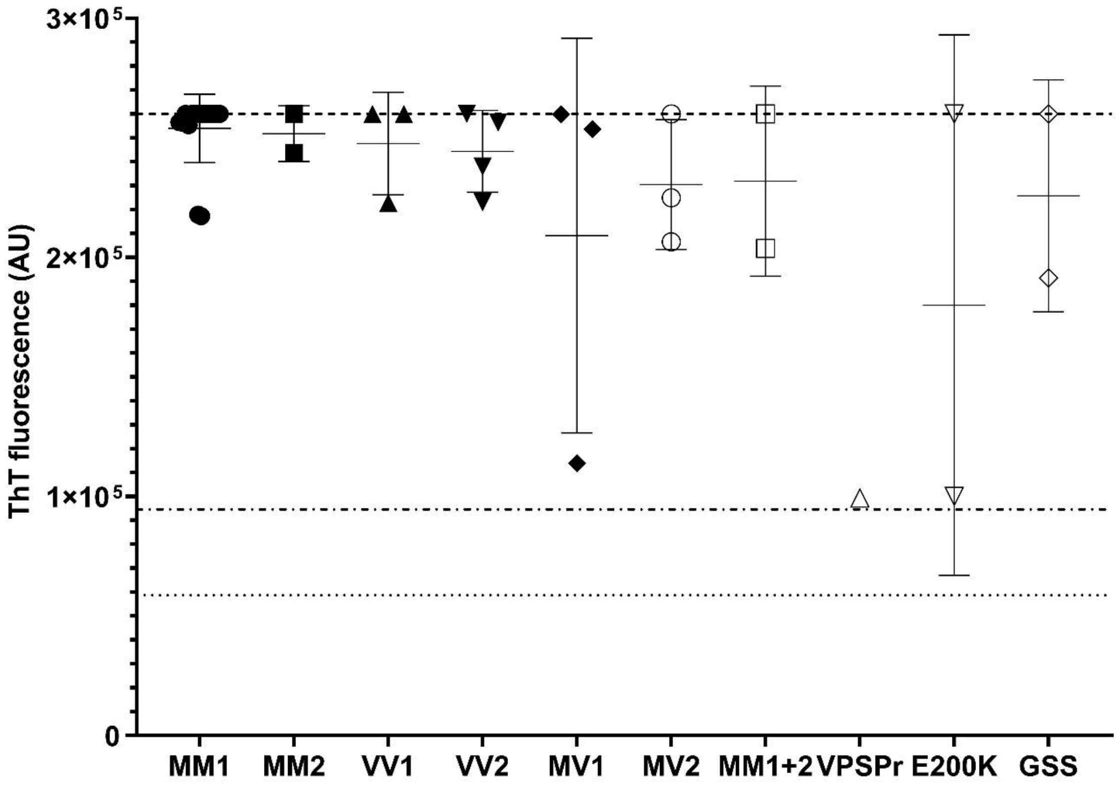

Mean maximal ThT fluorescence of the individual TSE patient *post-mortem* CSF samples analyzed by RT-QuIC and grouped according to the disease type. The size of the groups varied from n = 16 for CJD MM1 to n = 1 for VPSPr and the description of groups is present in Table 1. The points represent mean of maximal ThT fluorescence calculated from quadruplicate samples of individual patients. Error bars represent mean ± SD for the group of samples. The values are presented for analysis of undiluted CSF samples (n=30) and diluted CSF samples (n=8) for the samples which were negative undiluted. The dotted line and dash-dotted line represent the threshold value for undiluted and diluted samples, respectively. Dashed line depicts the upper limit of the fluorescence measurement. AU - arbitrary fluorescence units. Error bars represent mean ± SD for the group of samples.

Figure S5

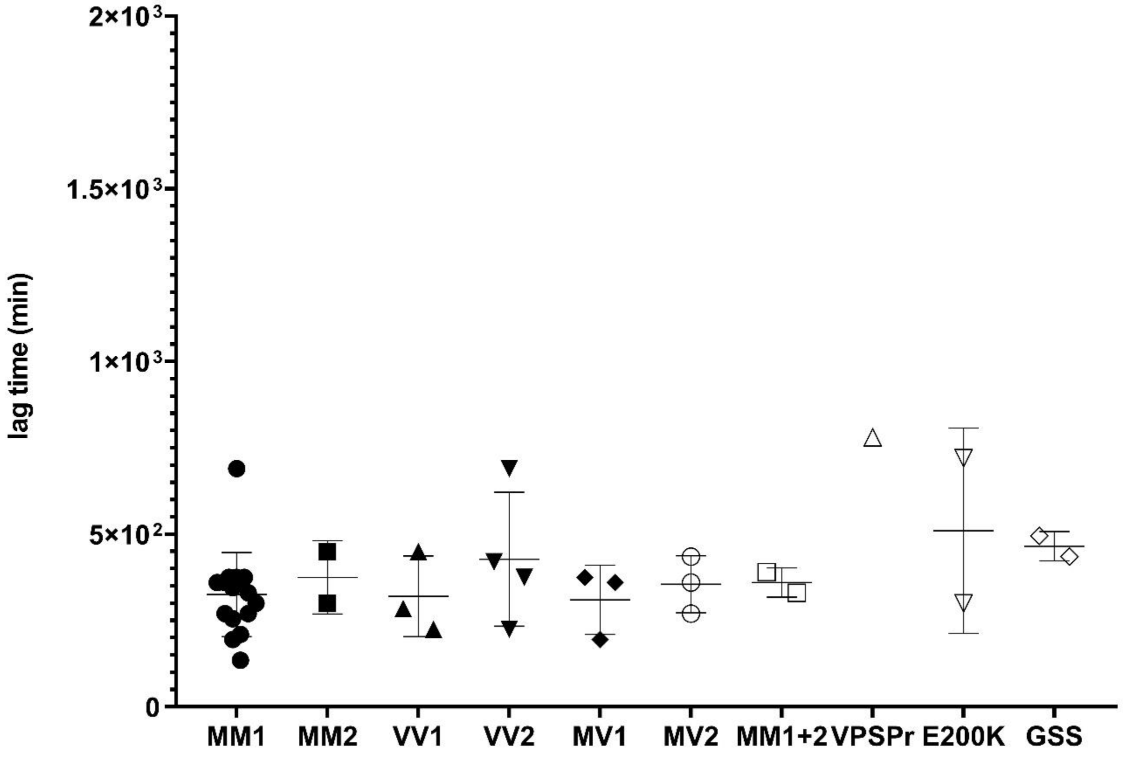

Length of lag time of *post-mortem* CSF samples of individual TSE patients analyzed by RT-QuIC and grouped according to the disease type. The size of the groups varied from n = 16 for sCJD MM1 to n = 1 for VPSPr. The description of the groups is present in Table 1. The points represent mean time needed for the fluorescence signal to cross the threshold value. The means were calculated from quadruplicate samples of individual patients. Error bars represent mean ± SD for the group of samples. The values are presented for analysis of undiluted CSF samples (n=30) and diluted CSF samples (n=8) for the samples which gave low signal undiluted. Error bars represent mean ± SD for the group of samples.

Figure S6

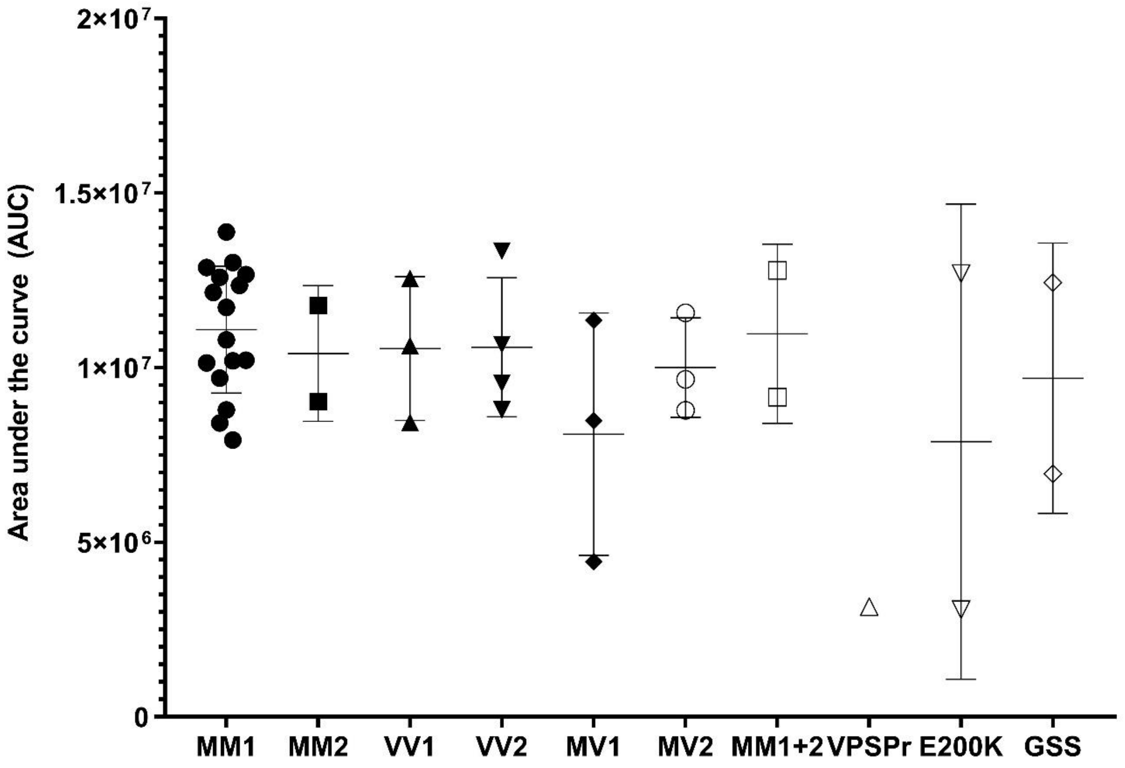

Area under the curve of *post-mortem* CSF samples of individual TSE patients analyzed by RT-QuIC and grouped according to the disease type. The size of the groups varied from n = 16 for sCJD MM1 to n = 1 for VPSPr. The description of the groups is present in Table 1. The points represent area under the curve of mean fluorescence signal trace calculated from quadruplicate samples of individual patients. Error bars represent mean ± SD for the group of samples. The values are presented for analysis of undiluted CSF samples (n=30) and diluted CSF samples (n=8) for the samples which gave low signal undiluted. Error bars represent mean ± SD for the group of samples.

Figure S7

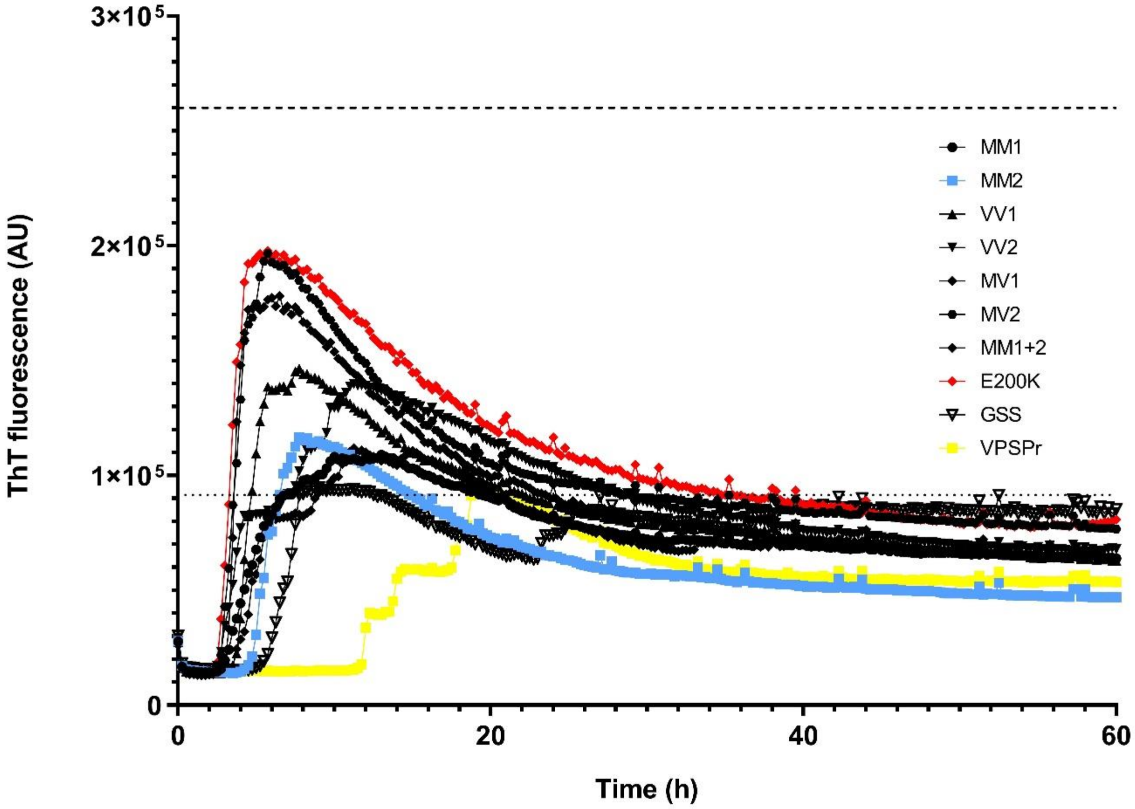

RT-QuIC time course of the mean ThT fluorescence of TSE *post-mortem* skin samples grouped according to the disease type. The size of the groups varied from n = 16 for CJD MM1 to n = 1 for VPSPr and the description of groups is present in Table 1. The traces represent mean fluorescence of all samples in the group. The dotted line represents the threshold value and dashed line depicts the upper limit of the fluorescence measurement. AU - arbitrary fluorescence units.

Figure S8

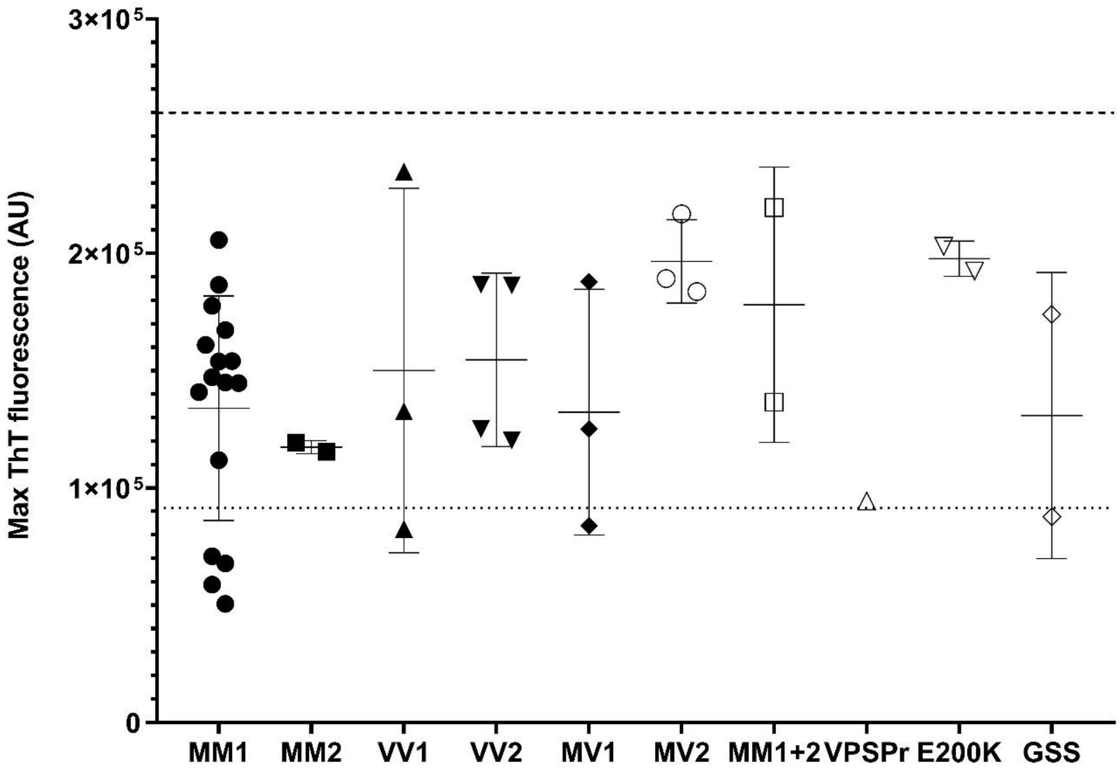

Mean maximal ThT fluorescence of the individual TSE patient *post-mortem* skin samples analyzed by RT-QuIC and grouped according to the disease type. The size of the groups varied from n = 16 for CJD MM1 to n = 1 for VPSPr and the description of groups is present in Table 1. The points represent mean of maximal ThT fluorescence calculated from quadruplicate samples of individual patients. Error bars represent mean ± SD for the group of samples. The dotted line represents the threshold value and the dashed line depicts the upper limit of the fluorescence measurement. AU - arbitrary fluorescence units. Error bars represent mean ± SD for the group of samples.

Figure S9

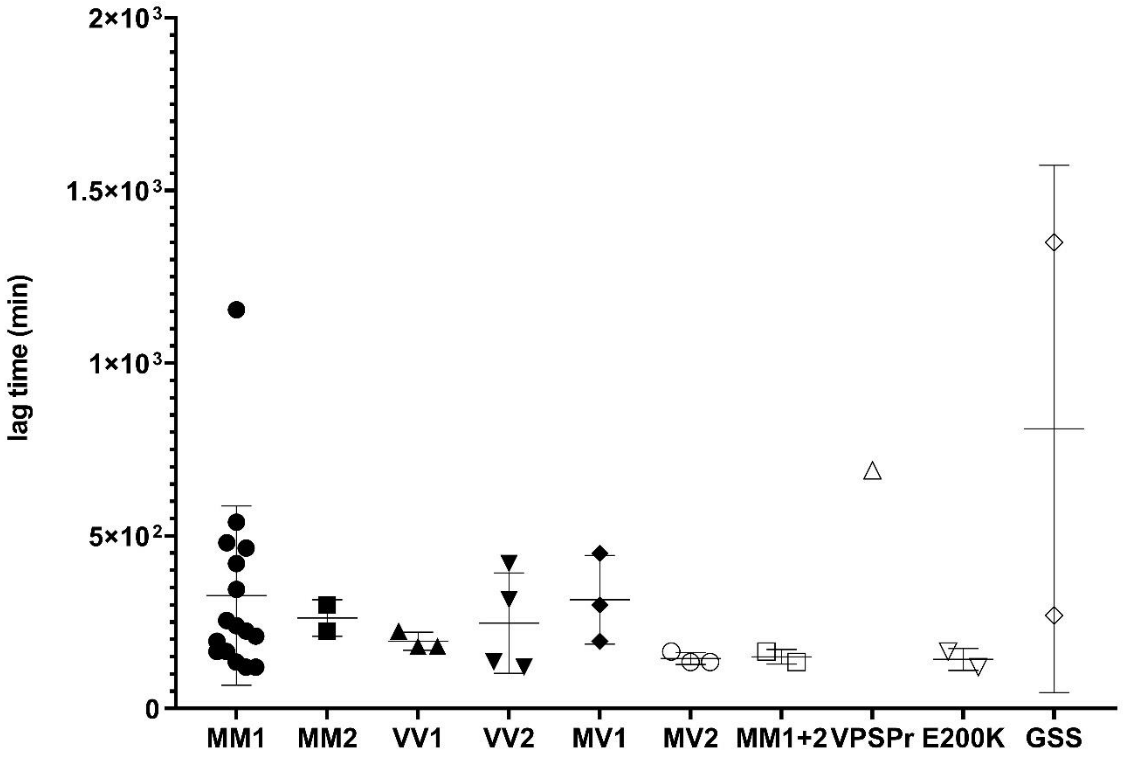

Length of lag time of *post-mortem* skin samples of individual TSE patients analyzed by RT-QuIC and grouped according to the disease type. The size of the groups varied from n = 16 for sCJD MM1 to n = 1 for VPSPr. The description of the groups is present in Table 1. The points represent mean time needed for the fluorescence signal to cross the threshold value. The means were calculated from quadruplicate samples of individual patients. Error bars represent mean ± SD for the group of samples.

Figure S10

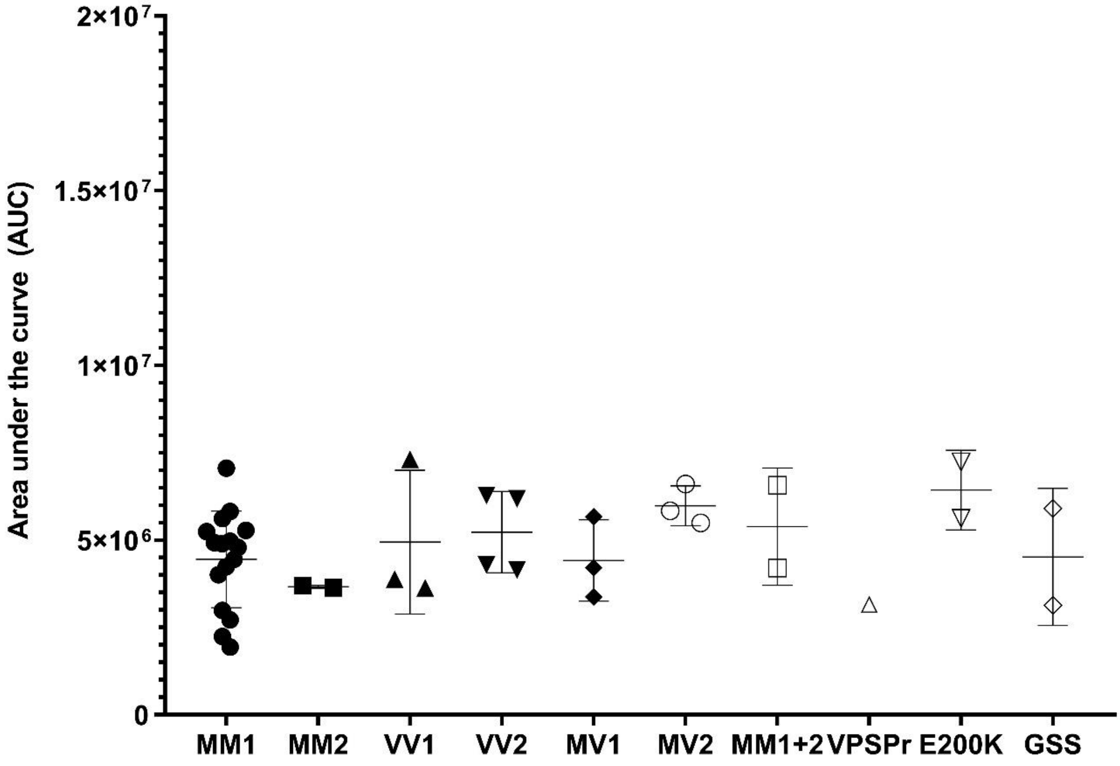

Area under the curve of *post-mortem* skin samples of individual TSE patients analyzed by RT-QuIC and grouped according to the disease type. The size of the groups varied from n = 16 for sCJD MM1 to n = 1 for VPSPr. The description of the groups is present in Table 1. The points represent area under the curve of mean fluorescence signal trace calculated from quadruplicate samples of individual patients. Error bars represent mean ± SD for the group of samples.

Figure S11

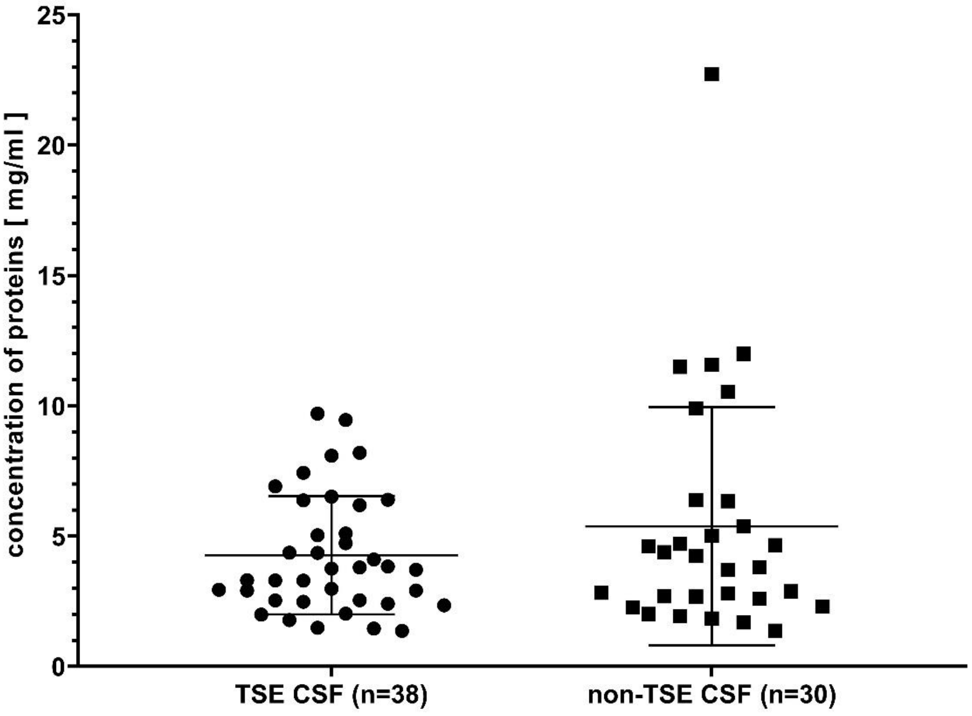

Protein content of individual TSE and control non-TSE CSF samples. The protein concentration was estimated using BCA assay. Error bars represent mean ± SD for the group of samples.

Figure S12

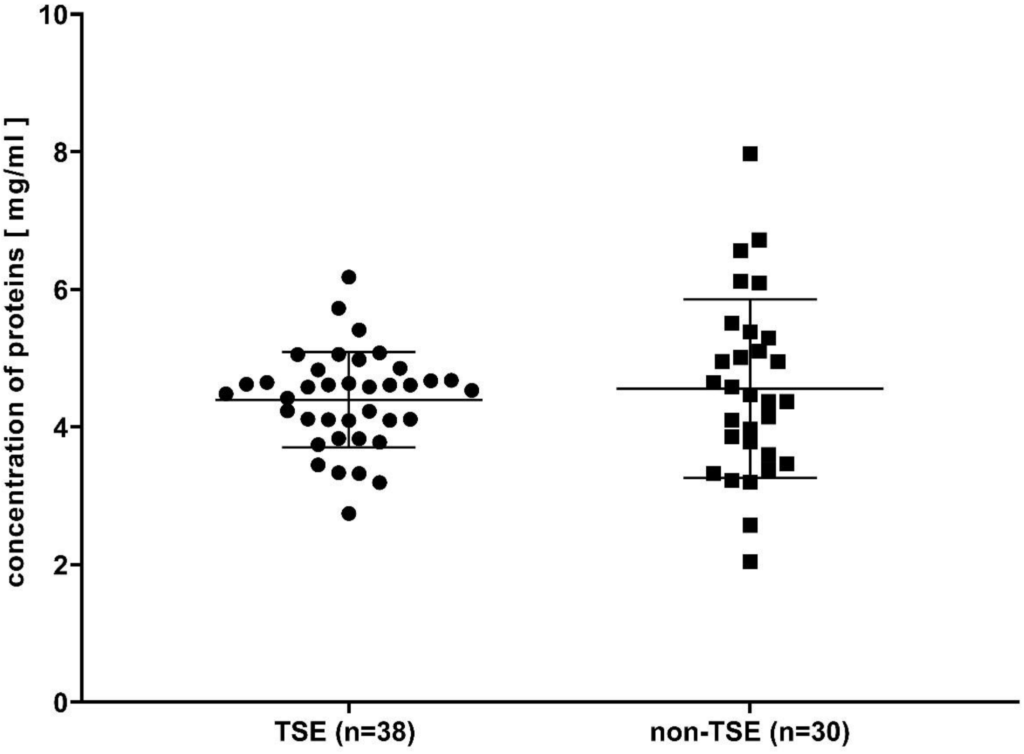

Protein content of individual TSE and control non-TSE skin homogenate samples. The protein concentration was estimated using BCA assay of collagenase skin homogenates used to seed RT-QuIC reaction. Error bars represent mean ± SD for the group of samples.

Figure S13

a)

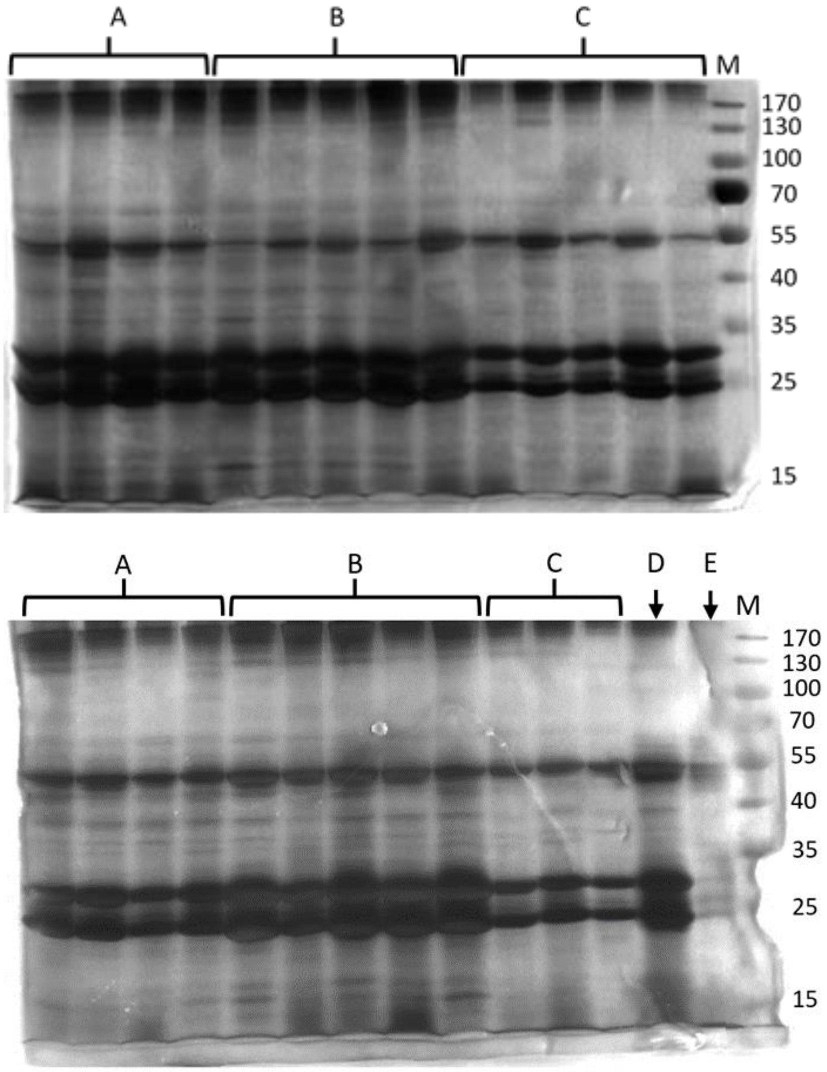

Protein pattern of skin homogenate supernatants used to seed RT-QuIC reactions. Skin homogenate samples of a) TSE (n=14) and b) control non-TSE (n=14) patients were analyzed using SDS PAGE on 10% gels and gels stained by silver. Representative homogenates with A) intermediate (~ 4 mg/ml), B) high (5-7 mg/ml) and C) low (2-3 mg/ml) protein content. Sample with highest D) and lowest E) protein content. M - molecular weight standard (kDa).
